# Supplementary material for: Injury epidemiology and emergency department length of stay in trauma hospital in Addis Ababa, Ethiopia
Source: PLoS One. 2024 Nov 11;19(11):e0309962. doi: 10.1371/journal.pone.0309962 (PMC11554118; doi:10.1371/journal.pone.0309962)
Supplement: Attachment — Submitted filename: Point-by-point response to the reviewers questions.docx [file pone.0309962.s001.docx]

**Point by point response to the reviewers**

We would like to thank the reviewer for the time and effort put on to revise our manuscript in detail. We believe that the comments have identified important areas which required improvement. After completion of the suggested edits, the revised manuscript has benefitted from an improvement in the overall presentation and clarity.

| **Comments (issues raised)** | **Response** |
| --- | --- |
| Comments from Review of #1 | Dear Reviewers thank you for your comment and support through the process. The following points are the response to your idea. |
| 1. The manuscript has a lot of editorial issues (incomplete ideas, repeated words, grammar, punctuation etc…) | **Accepted the comment:**  1. Thank you for the critical observation. We made a thorough edition throughout the document. |
| 2. Please be consistent when you present your findings for example using both the frequency and (percentage) or only the percentage | **Accepted the comment** |
| 3. The authors mention that “neither the 210 Injury Severity Score nor the Revised Trauma Scale was used to determine severity”. if that is the case, what specific criteria do they use to label yellow/green, orange, and red categories? | **Thank you for the critical observation. We agreed in your issues**  The Trauma Hospital used color coding for departmentalizing the emergency department (ED) room. The ED has four zones (or wards) to treat patients according to the Emergency Severity Index scale (1). These zones are identified by color-coded triage zones, which include green, yellow, orange, and red. In line with the WHO's classification of injury (2), which categorizes injuries as yellow/green (mild), orange (requires skilled care and treatment), and red (requires intensive medical/surgical care). The severity is believed to increase from the green to the red zone along the color spectrum. We used secondary data with incomplete data in the form of Injury Severity Score or the Revised Trauma Scale. However, we categorized based on severity according to the Emergency Severity Index scale of color coding. |
| 4. I am not clear on the objective of this manuscript the outcome of this study was the length of stay at ED. The independence variables were sociodemographic variables (sex, age, and residence), triaged zone, mechanisms of injury, affected body parts, and nature of injuries. All the mentioned independent variables are linked with the patient, not the facility and the service. But in most of the cases, the length of stay in the ED depends on the capacity of the facilities, the service, and infrastructure-related problems and this needs additional investigation using a qualitative approach. | **Thank you for the critical observation. We agreed in your issues.**    This study aimed to describe injuries and identify factors associated with patients' prolonged lengths of stay (LoS) in the Emergency Department (ED). The findings of this study are based on a large dataset and provide information about the magnitude of injury burden, characteristics of injury in individuals, mechanism, and outcome. LoS was analyzed based on existing data due to record availability. However, we acknowledge the limitations of our study and recommend further research in a broader context based on primary data. |
| 5. The authors mention that the mean (SD) length of patients who stayed in the emergency department was 2.51 (5.18) with a range of 1 to 181 days. Why do they stay at the ED for a long time? They should be transferred either to a different ward or to other facilities for better care. According to Ethiopian hospital guidelines, patients requiring emergency services should be kept at the ED for a maximum of 24 h. | **Thank you for the critical observation.** We reviewed your concerns and have used the existing records. However, we also cross-checked the data with physicians and other workers in the ED. The reasons why patients stay for a long period are as follows:  1. Patient load in other departments, which leads to unavailability of beds.  2. Shortage of diagnostic tools.  3. There were patients without a responsible proxy or family members. These patients remained in the ED even after completing their treatment until a nonprofit /charity organization agreed to provide shelter and food support outside of the hospital. |
| 6. I think the study should be modified to the length of stay at the facility, not at the ED | **Thank you for the critical observation**.  However, we emphasized the burden of patients in the ED, not in all departments in the hospitals. This is because the ED is the first area where lives are saved through appropriate support and linking to the right services at the right department. |

1.	Ayano WA, Fentie AM, Tileku M, Jiru T, Hussen SU. Assessment of adequacy and appropriateness of pain management practice among trauma patients at the Ethiopian Aabet Hospital: A prospective observational study. BMC emergency medicine. 2023;23(1):92.

2.	The World Health Organization. Injury Survelance Guidelines Switzerland2001 [02-11-2023]. Available from: https://[www.who.int/publications/i/item/924159133](http://www.who.int/publications/i/item/924159133).
